# Post-translational control of RIPK3 and MLKL mediated necroptotic cell death

**DOI:** 10.12688/f1000research.7046.1

**Published:** 2015-11-19

**Authors:** James M. Murphy, James E. Vince

**Affiliations:** 1 The Walter and Eliza Hall Institute of Medical Research, Parkville, Victoria, Australia; 2 Department of Medical Biology, University of Melbourne, Parkville, Victoria, Australia

**Keywords:** necroptotic cell death, necroptosis, RIPK3, MLKL, mixed lineage kinase domain-like, receptor interacting protein kinase-3

## Abstract

Several programmed lytic and necrotic-like cell death mechanisms have now been uncovered, including the recently described receptor interacting protein kinase-3 (RIPK3)-mixed lineage kinase domain-like (MLKL)-dependent necroptosis pathway. Genetic experiments have shown that programmed necrosis, including necroptosis, can play a pivotal role in regulating host-resistance against microbial infections. Alternatively, excess or unwarranted necroptosis may be pathological in autoimmune and autoinflammatory diseases. This review highlights the recent advances in our understanding of the post-translational control of RIPK3-MLKL necroptotic signaling. We discuss the critical function of phosphorylation in the execution of necroptosis, and highlight the emerging regulatory roles for several ubiquitin ligases and deubiquitinating enzymes. Finally, based on current evidence, we discuss the potential mechanisms by which the essential, and possibly terminal, necroptotic effector, MLKL, triggers the disruption of cellular membranes to cause cell lysis.

## Introduction

### RIPK3 and MLKL are essential for necroptotic cell death

Necroptosis is a caspase-independent programmed cell death pathway ^1^. Necroptotic cell lysis and the release of intracellular pro-inflammatory molecules is dependent on phosphorylation of the pseudokinase, MLKL, by the protein kinase, RIPK3. Phosphorylation of MLKL leads to its activation, and subsequent cell death by mechanisms that are currently a matter of debate, although a weight of evidence suggests this involves disruption of cellular membranes, including the plasma membrane. Following tumor necrosis factor (TNF) receptor 1 (TNFR1) ligation, the protein kinase, RIPK1, activates RIPK3 presumably by promoting RIPK3 autophosphorylation, which in turn leads to MLKL activation. However, it is now apparent that RIPK3 can be activated independently of RIPK1 in many circumstances, and that RIPK1 can act to repress RIPK3 activation, both *in vitro* and *in vivo* ^2– 8^. Consequently, our current thinking is that RIPK3 and MLKL are the core machinery essential for all necroptotic cell death responses.

### RIPK3 is a driver of MLKL dependent and independent inflammatory disease

Studies using RIPK3-deficient mice have implicated pathological RIPK3 signaling, and potentially necroptosis, in many inflammatory diseases, such as atherosclerosis, kidney ischemia reperfusion injury, liver injury, myocardial infarction, and multiple sclerosis (reviewed in 9). However, it has recently been posited that necroptosis may also act in an anti-inflammatory capacity, as cell death abrogates TNF- or toll-like receptor- (TLR) induced transcription of pro-inflammatory cytokines and the ensuing inflammatory response ^10^. Recent research has also revealed that RIPK3 has several non-necroptotic signaling capabilities, both *in vitro* and *in vivo* (reviewed in 11). These include the ability to activate caspase-8 dependent apoptosis ^12– 14^, trigger interleukin-1β (IL-1β)-dependent inflammation through caspase-8 and/or the Nod-like receptor 3 (NLRP3) inflammasome ^15– 22^, and regulate the transcription of cytokines ^23, 24^. Hence, the use of MLKL-deficient mice is required to validate necroptosis as a *bona fide* drug target in many inflammatory disease models where RIPK3 has been implicated. In this regard, murine genetic studies have started to document how unrestrained MLKL-dependent necroptotic signaling can result in embryonic lethality ^3^ and cause liver inflammation ^25^. In addition, the development of phospho-specific MLKL antibodies as markers of activated MLKL have shown that necroptosis is likely to occur in diseases such as toxic epidermal necrolysis ^26, 27^, drug-induced liver injury ^28^, and pathogen infection ^21^. Cancer cell lines have also been observed to suppress RIPK3 expression ^29^, which in some circumstances has been attributed to DNA methylation ^30^. As such, chemically induced hypomethylation can restore RIPK3 expression and promote RIPK3-MLKL-induced necroptosis in response to chemotherapeutic treatments. A greater understanding of the mechanisms of necroptosis signaling, and when it occurs, is therefore likely to yield new therapeutic opportunities in a number of different disease states.

### Necroptosis is activated by a number of different receptors

Several signaling receptors have been documented to activate RIPK3-MLKL dependent necroptosis, including death receptors (i.e., TNFR1), TLRs, the DNA receptor DAI (DNA-dependent activator of interferon [IFN]-regulatory factors or ZBP1/DML-1), and the T-cell antigen receptor. Type I IFN and IFNγ-induced transcriptional responses have also been proposed to cause necroptosis, or to enhance TLR3/4 and TNFR1 necroptosis ^31– 33^. While protein kinase R (PKR) was suggested to directly trigger formation of the RIPK1-RIPK3 necrosome downstream of IFNγ signaling ^33^, PKR is not required for type I IFN killing ^32^, and hence the underlying mechanism for IFN-induced necroptosis requires further study. By comparison, necroptotic signaling caused by TNFR1 ligation is better defined (reviewed in 34). In most cases, TNF binding to TNFR1 triggers the formation of a cell surface complex, complex I, that induces the transcription of pro-survival genes and inflammatory cytokines. Mechanistically, the death domain (DD) of TNFR1 interacts with the DD of TNFR1-associated death domain (TRADD) (and potentially the DD of RIPK1) to nucleate the formation of a large multimeric TRADD-RIPK1-TRAF2- inhibitor of apoptosis (IAP) ubiquitylation platform ^35– 38^. For example, RIPK1 binding to this complex and its modification with ubiquitin chains by IAP proteins parallels IAP-dependent recruitment of the linear ubiquitin chain assembly complex (LUBAC). Ubiquitylated RIPK1, and LUBAC modification of NEMO (nuclear factor kappa-light-chain-enhancer of activated B cells [NFκB] essential modifier), subsequently activate canonical NFκB signaling. In the absence of optimal RIPK1 ubiquitylation (i.e., when IAPs are lost), RIPK1 dissociates into the cytosol to form a secondary death-inducing complex that can activate caspase-8 (complex II) to cause apoptosis. Caspase-8 represses necroptotic signaling, and hence, when caspase-8 activity is low, RIPK1 can bind RIPK3 to form the necrosome, activate MLKL, and induce necroptotic killing.

### Physiological triggers of necroptosis

Because under normal cell culture conditions necroptosis is not induced by death receptor or TLR ligation, experimentally, necroptosis is usually studied by deleting or inhibiting key negative regulators of necroptotic signaling, such as caspase-8 or IAP proteins (see below). Physiological settings that trigger necroptosis have been less well defined, although situations where caspase-8 is down-regulated, such as following cutaneous wounding ^39– 41^, or IAP protein depletion, such as during TNF-like weak inducer of apoptosis (TWEAK)-FGF-inducible molecule 14 (FN14) TNF superfamily signaling ^42, 43^, clearly have the potential to promote a necroptotic response. Along these lines of evidence, biopsies from children suffering from inflammatory bowel disease display decreased caspase-8 expression and elevated RIPK3 and MLKL levels, and may indicate ongoing necroptosis ^44^. More direct experiments have been performed to suggest that bacterial and viral molecules can act to induce or inhibit necroptosis at multiple levels, including direct RIPK1/RIPK3 targeting, or downstream of MLKL phosphorylation ^21, 45– 50^.

In this review we summarize recent advances in identifying and understanding positive and negative regulators of necroptotic signaling ( Table 1), focusing on findings with strong genetic evidence. We begin by a brief discussion on the best studied necroptotic components; RIPK1, RIPK3 and MLKL.

**Table 1.** Post-translational modification of RIPK1, RIPK3 and MLKL.

Protein	Target residue	PTM	Writer	Eraser	Impact of PTM	References
Mouse RIPK1	S89	Putative phosphorylation			Putative pS89; suppresses necroptosis in Jurkat cells	113
Mouse/Human RIPK1	K377 and others	K63-, K48-, K11- linked ubiquitylation	cIAP1/ cIAP2	A20	Ubiquitination prevents death signalling	103, 114– 118
						
Mouse RIPK3	S204	Phosphorylation			pS204 promotes necroptosis	113
Human RIPK3	S199	Phosphorylation	RIPK3		pS199 promotes necroptosis	119
						
Mouse RIPK3	T231/S232	Phosphorylation		Ppm1b	pT231/pS232 promotes MLKL interaction and necroptosis	101, 102, 113
Human RIPK3	S227	Phosphorylation			pS227 promotes MLKL interaction and necroptosis	57
						
Mouse RIPK3	K5	Ubiquitylation		A20	K5Ub promotes necrosome assembly and necroptosis	15, 96
Mouse RIPK3	C119	S-nitrosylation			Promotes necroptosis	120
						
Mouse MLKL	S345, S347, T349	Phosphorylation	RIPK3		pS345 and pS347 promote necroptosis	60, 64, 65
						
Human MLKL	T357, S358	Phosphorylation	RIPK3		pT357/pS358 promote necroptosis	57
						
Mouse MLKL	S124	Phosphorylation			Nil. Counterpart of human S125	65, 121
Mouse MLKL	S158	Phosphorylation			pS158 likely suppresses necroptosis	65
Mouse MLKL	S228	Phosphorylation	RIPK3		pS228 relieves possible RIPK3-mediated suppression of necroptosis	65
Mouse MLKL		Ubiquitylation			Unknown. Correlates with necroptosis	15

## The core necroptotic machinery

### RIPK3

The defining feature that triggers RIPK3 serine/threonine kinase activity and its phosphorylation of MLKL is RIPK3 oligomerization *via* RIP homotypic interaction motif (RHIM) containing proteins. For example, RHIM containing signaling components of TNFR1 (RIPK1), TLR3 and TLR4 (TRIF) and DAI itself can all form RHIM-RHIM interactions with RIPK3 to trigger necroptotic death. Similarly, the artificial dimerization/oligomerization of RIPK3 directly suffices to cause its activation and recruitment of MLKL in the absence of any upstream signal ^4, 14, 51^. The importance of RHIM-RHIM interactions for RIPK3 signaling is highlighted by several viral RHIM-containing proteins, such as cytomegalovirus vIRA and herpes simplex virus ICP-6 and ICP-10, which bind RIPK1 and RIPK3 and can regulate necroptosis to alter host anti-viral responses ^46– 49^.

The interaction of RIPK1 and RIPK3 RHIMs induces the formation of a large amyloid-like necrosome signaling platform ^52^. Recent work has suggested that RIPK1-RIPK3 heterodimers do not suffice to trigger necroptosis, and instead RIPK3 homo-oligomer formation within the necrosome complex is essential for RIPK3 activation, intramolecular RIPK3 phosphorylation, and MLKL recruitment ^51^. This model fits with recent genetic studies showing that the deletion of RIPK1 *in vivo* can trigger RIPK3-MLKL induced necroptosis ^3^, as the loss of RIPK1 may enhance the propensity for RIPK3 oligomerization ^4^.

Activated RIPK3 phosphorylates MLKL, thereby allowing MLKL association with, and disruption of, phospholipid membranes ^28, 53– 55^. RIPK3-deficient mice display no overt phenotype, and hence targeting RIPK3 kinase activity with small-molecule inhibitors represents one strategy for the treatment of necroptotic-driven diseases ^13, 56^. It is noteworthy, however, that RIPK3 kinase inhibition ^13^, or a D161N mutation in the RIPK3 kinase domain *in vivo* ^12^, can drive lethal caspase-8-dependent apoptosis by triggering RIPK1 recruitment to RIPK3, and RIPK1-mediated activation of caspase-8. Interestingly, RIPK3 kinase activity *per se* is not essential for mammalian viability, as an independently generated kinase-dead (K51A) RIPK3 mouse is viable and fertile ^13^. Hence, the development of RIPK3 kinase inhibitors that avoid lethal RIPK3 conformational changes are required. Alternatively, MLKL inhibitors ^54, 57^ may be a more attractive strategy for specifically targeting necroptosis, as these would avoid complications that may result from altering non-necroptotic RIPK3 signaling capabilities.

### MLKL

**Figure 1.**
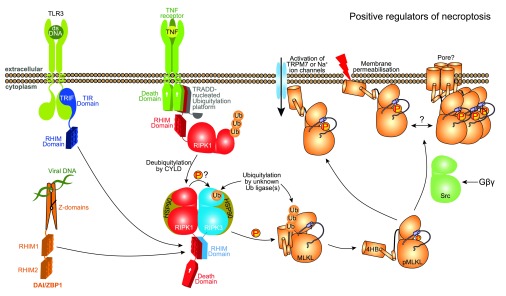
Positive regulators of necroptosis. The core necroptosis machinery, comprising RIPK3 and MLKL, are activated following RIPK3 interaction with RIPK1, TRIF or DAI *via* their RHIMs. CYLD-mediated deubiquitylation of RIPK1 is necessary for its participation in cell death pathways, while ubiquitylation of RIPK3 (at Lys5) and MLKL by as-yet-unidentified E3 ligases may promote necroptosis ^15, 97^. HSP90 is known to augment the necroptotic functions of RIPK1 and RIPK3. MLKL is believed to induce cell death *via* a membrane-directed process ^54^, perhaps by directly permeabilizing membranes ^28, 55^, with some debate over whether channel activation might be involved ^28, 53, 63^, or, as one report suggests, there may be a role for Src in promoting MLKL-mediated death downstream of Gβγ. Abbreviations: 4HB, four-helix bundle; CYLD, cylindromatosis; DAI, DNA-dependent activator of interferon [IFN]-regulatory factors; HSP90, heat shock protein 90; IAP, inhibitor of apoptosis proteins; MLKL, mixed lineage kinase domain-like; RHIM, RIP homotypic interaction motif; RIPK, receptor interacting protein kinase; TLR, toll-like receptor; TNF, tumour necrosis factor; TNFR1, TNF receptor 1; TRADD, TNFR1-associated death domain; TRAF2, TNF receptor associated factor 2; TRPM7, transient receptor potential cation channel, subfamily M, member 7.

MLKL is an essential necroptosis effector that operates downstream of RIPK3 ^57– 60^. Unlike RIPK3, which is a catalytically active, conventional protein kinase, MLKL belongs to a group of related proteins that are enzymatically-dead, termed pseudokinases ^61, 62^. In addition to its C-terminal pseudokinase domain, MLKL contains an N-terminal four-helix bundle (4HB) domain, which is now known to mediate MLKL’s killer function ^53– 55, 63^. Our current understanding of MLKL activation is that RIPK3 phosphorylates the activation loop in MLKL’s pseudokinase domain ^57, 59, 60, 64, 65^, which induces a conformational change that relieves the suppressive function of the pseudokinase domain, allowing the unleashing of the executioner N-terminal 4HB domain ^54^. Several different models for how the MLKL 4HB domain can induce cell death have been proposed, ranging from a direct membrane permeabilization or pore-forming capacity ^28, 55, 66^, to the implication of downstream effectors ^53, 63^ (summarized in Figure 1). Although details are still emerging, recombinant MLKL was found to bind to various lipids in “fat Western” lipid arrays ^28, 55^, which was further reflected in a capacity to permeabilize liposomes *in vitro* ^28, 55, 66^. Curiously, MLKL most readily permeabilized liposomes containing 15% cardiolipin, a lipid believed to be confined to the mitochondrial inner membrane. The necessity of mitochondria for necroptosis has been challenged ^67^, making the preference for cardiolipin difficult to reconcile. At the molecular level, the mode of 4HB domain engagement of lipids remains of outstanding interest. While initial reports suggested this interaction might be mediated by positively-charged residues in the human MLKL 4HB domain ^55^, these residues are poorly conserved among MLKL orthologs and indeed, curiously, mutation of the acidic counterparts in the mouse 4HB domain similarly compromised the capacity of the domain to induce cell death ^54^. It is therefore possible that, rather than a direct interaction with negatively-charged phospholipids, these charged residues in the MLKL 4HB domain are important to the MLKL homo- and/or hetero-oligomerisation that is necessary for necroptosis.

**Figure 2.**
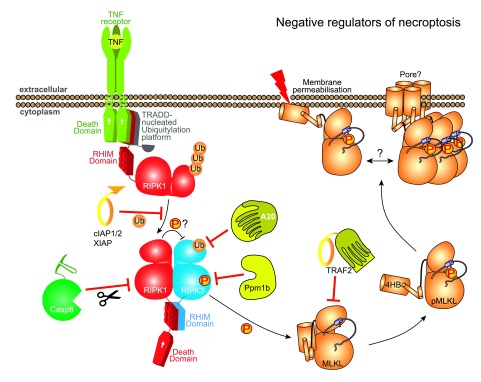
Negative regulators of necroptosis. Negative regulation at the levels of RIPK1, RIPK3 and MLKL have been reported to attenuate necroptotic signalling. Not only is necroptosis by definition a caspase-independent process, but caspase-8 negates cell death, potentially by proteolytically-cleaving CYLD, RIPK1 and RIPK3. Reversible tuning of the pathway is accomplished by introduction or removal of post-translational modifications, including: ubiquitylation of RIPK1 by IAPs; deubiquitylation of RIPK3 on Lys5 by A20; and dephosphorylation of mouse RIPK3 at S231/T232 by Ppm1b. Whether an analogous phosphatase exists to dephosphorylate activated MLKL and inhibit necroptosis is not clear. However, other regulators of MLKL have been proposed, such as TRAF2. It is currently unknown what factors might govern phospho-MLKL assembly into higher order oligomers on the membrane, although a role for additional factors in mediating death is suggested by the lag between MLKL membrane translocation/oligomerisation and cell death ^65^. Abbreviations: 4HB, four-helix bundle; CYLD, cylindromatosis; IAP, inhibitor of apoptosis proteins; MLKL, mixed lineage kinase domain-like; RHIM, RIP homotypic interaction motif; RIPK, receptor interacting protein kinase; TNF, tumour necrosis factor; TRADD, TNFR1-associated death domain; TRAF2, TNF receptor associated factor 2.

Another outstanding question is whether the MLKL 4HB domain forms a transmembrane pore or merely somehow compromises the integrity of the membrane. In concert with a greater understanding of which membrane within a cell is the target of MLKL action, knowledge of the means by which MLKL breaches membranes is essential for a comprehensive understanding of its action. Even though the membrane compromising activity of recombinant MLKL in liposome assays is highly suggestive of MLKL acting as a lone gunman, several lines of evidence have implicated other proteins as either co-effectors that augment or negate the activities of RIPK3 or MLKL, or as effectors that function downstream of MLKL activation ( Figure 1 and Figure 2). Of note, the induction of necroptosis is associated with the ubiquitylation of RIPK3 and MLKL ^15^, although the function of these modifications requires further study. We have summarized important necroptotic regulators that promote ( Figure 1) or negate ( Figure 2) MLKL-mediated death, and elaborate on current knowledge of their activities below.

### RIPK1

An important role for RIPK1 and its kinase activity in death receptor-induced necroptosis was documented long before RIPK3 was identified as the essential necroptotic RIPK1 binding partner ^68^. The development of the RIPK1 kinase inhibitor necrostatin-1 ^69, 70^ has been widely used to demonstrate the therapeutic potential of targeting RIPK1 kinase activity and necroptosis in disease models. The plausibility of this strategy was recently validated when, in contrast to the embryonic lethality of RIPK1-deficient mice caused by unrestrained apoptotic and necroptotic signaling ^2, 3, 5^, RIPK1 kinase-dead knockin mice, containing K45A ^2^ or D138N ^12, 71^ alleles, were shown to be viable and fertile. Hence, while RIPK1 kinase activity is often a requisite for necroptotic killing, it is not critical for mammalian survival. Instead the kinase-independent scaffolding role of RIPK1 is vital in preventing lethal RIPK3-MLKL and caspase-8 signaling.

Mechanistically, RIPK1 phosphorylation of RIPK3 is an attractive hypothesis for why RIPK1 kinase function is required for TNF-induced necroptosis. However, RIPK1 mediated phosphorylation of RIPK3 has not been formally reported to date, and the key target(s) of RIPK1 kinase activity remain unclear. Recently it has been suggested that the inhibition of RIPK1 kinase activity enhances RIPK1’s anti-necroptotic function, and consistent with this, depletion of total RIPK1 in several cell types was shown to sensitize to TNF and TLR-induced necroptotic killing ^7^. Why RIPK1 loss sensitizes some cell lines to TNF-induced necroptosis, while in others it confers protection, may be due to the expression levels of RIPK3 in different cells or tissues, dictating its ability to efficiently engage TNFR1 or TLR complexes and form RIPK3 oligomers. Consistent with this notion, the expression levels of RIPK3 have been shown to largely determine whether TNF can engage RIPK3 killing in the absence of RIPK1 ^72^. Unlike the direct recruitment of RIPK3 to the RHIM containing receptor DAI ^46^, or the TLR adaptor protein TRIF ^56, 73^, the way RIPK3 is recruited to TNF receptor complexes in RIPK1 deficient cells is unknown.

## Positive regulators of necroptosis

### CYLD

CYLD (Cylindromatosis) is a deubiquitinating enzyme that removes K63-linked and linear ubiquitin chains from its target proteins, which include RIPK1 and TRAFs. This deubiquitinase activity of CYLD is linked with increased TNF-induced death signaling, including RIPK1-RIPK3 necrosome formation and necroptotic cell death ^74– 76^. Mechanistically, CYLD has been proposed to deubiquitylate RIPK1 during necrosome complex formation to facilitate RIPK1/3 kinase activity ^76^. In this context, it is interesting to note that the LUBAC component, HOIL-1 interacting protein (HOIP), can ubiquitylate RIPK1 within the necrosome ^77^. Although HOIP loss did not impact necroptotic killing, the functional consequences of necroptotic-associated RIPK1, RIPK3 and MLKL ubiquitylation warrant further investigation.

Following TNF stimulation, aberrant CYLD necroptotic activity is held in check by caspase-8 mediated CYLD processing and inactivation ^74^. Consistent with this, colitis induced by deletion of the essential caspase-8 adaptor protein Fas-associated protein with death domain (FADD) in intestinal epithelial cells was ameliorated by loss of either RIPK3 or CYLD activity ^78^. CYLD is thereby important for necroptotic signaling in some situations *in vitro* and *in vivo*. In contrast, however, unlike RIPK3-deletion, the *in vivo* inactivation of CYLD delays, but does not prevent, the inflammation caused by unrestrained necroptotic killing of FADD deficient keratinocytes ^79^. Hence, although CYLD tunes necroptotic activity, it is not essential for it to occur in all cell types.

### HSP90

The Cdc37-heat shock protein 90 (HSP90) co-chaperone system has been widely implicated as a regulator of protein kinase “clients”. Conventionally, HSP90 interaction is thought to enhance protein stability, often through protection from proteasomal degradation, as is believed to be the case with RIPK1 ^80– 85^. RIPK3 has long been recognized as an HSP90 client protein ^86, 87^, although only recently has a less passive regulatory role been suggested ^88^. Inhibition of HSP90 or genetic knockdown of the kinase-interaction adaptor, Cdc37, inhibited RIPK3’s capacity to phosphorylate MLKL to induce necroptosis, but only conferred modest effects on RIPK3 abundance ^88^. The precise underlying mechanism remains a matter of outstanding interest.

### Channels

MLKL was implicated as a regulator of plasma membrane ion channels in two independent studies: one suggested a role for TRPM7 (transient receptor potential cation channel, subfamily M, member 7) in necroptotic killing ^53^, and another more broadly for Na ^+^ ion channel signaling ^63^. Subsequent studies using ion-free media, however, have challenged whether ion channels play an obligate role in mediating necroptosis ^28^, but instead suggest they contribute to varying extents in different cultured cell lines under some circumstances.

### Gβγ-Src signaling

A genetic screen for additional necroptosis effectors identified the transmembrane G-proteins, Gβ and Gγ, as instigators of an alternative pathway that operates in parallel with TNF-induced necroptosis ^89^. Perturbation of Gβγ signaling disrupted MLKL oligomerization and translocation to membranes, a finding attributed to perturbed activation of the protein kinase, Src. The mechanistic underpinnings and universality of this pathway have not yet been fully elucidated, but are illustrative of the many possible signaling effectors that could potentially intersect with and tune the necroptosis signaling pathway. This idea is supported by the recent identification and functional characterization of MLKL phosphorylation sites outside the best-understood phosphorylation sites within the MLKL pseudokinase domain activation loop ^65^. This study is illustrative of a broader potential role of post-translational modifications in modulating the activities of RIPK3 and MLKL.

## Negative regulators of necroptosis

### Caspase-8

Caspase-8-deficient embryonic lethality, or inflammatory disease resulting from tissue or cell type specific caspase-8 deletion, is rescued by RIPK3 or MLKL loss (reviewed in 9), demonstrating that caspase-8 is an essential repressor of potentially lethal necroptotic activity. Notably, this pro-survival function for caspase-8 does not require caspase-8 processing ^90^, but appears to be mediated by the catalytic activity of caspase-8/FLICE-inhibitory protein (cFLIP) heterodimers ^91^. The critical caspase-8 targets required to repress necroptosis remain unclear, although caspase-8 cleavage of key necroptotic inducers, including RIPK1 ^92^, RIPK3 ^93^, and CYLD ^74^, have been described and are likely to be important.

### IAP proteins

Mammalian IAP proteins are ubiquitin E3 ligases and include cIAP1, cIAP2 and XIAP (reviewed in 94). The cIAPs target RIPK1 for ubiquitylation to propagate TNF-induced NFκB activation and pro-survival responses, and also to prevent RIPK1 from associating with, and activating, caspase-8. XIAP, on the other hand, is a direct inhibitor of apoptotic caspases, although its deletion in mice ** does not result in an overt phenotype. In contrast, the co-deletion of cIAP1 and cIAP2 causes embryonic lethality, which is rescued in part by loss of RIPK1, RIPK3 or TNFR1 ^95^. Strikingly, while cIAP1/2 loss in combination with caspase inhibition is sufficient to trigger necroptosis in several cell types, in bone marrow ** derived macrophages and dendritic cells XIAP is more important for limiting TNF- and TLR-induced killing ^15, 19^. For example, XIAP loss alone can confer some sensitivity to TNF- and TLR-induced necroptosis and apoptosis, which is greatly enhanced by cIAP1/2 co-deletion ^15^. Lipopolysaccharide (LPS) treatment of wildtype macrophages triggers IAP-dependent RIPK3 ubiquitylation ^15^. Because LPS stimulation alone does not perturb macrophage viability, this may indicate that, akin to RIPK1 ubiquitylation, RIPK3 ubiquitylation under these conditions regulates transcriptional and/or pro-survival responses. However, LPS-induced necroptotic signaling caused by the loss of IAPs and caspase inhibition is also associated with increased RIPK3 (and MLKL) ubiquitylation ^15^, which in this case may play a pro-necroptotic role ^96^. At this stage the identity of the key XIAP substrate required to limit RIPK3-MLKL signaling remains unclear. It is possible that other ubiquitin E3 ligases also negatively regulate necroptosis signaling. Consistent with this, it has been suggested that the ubiquitin ligase MKRN1 (Makorin RING finger protein-1) represses RIPK1-RIPK3 complex formation, although how it does so is incompletely understood ^97^.

### TRAF2

TNF receptor associated factor 2 (TRAF2) binds to cIAP1/2 *via* a cIAP interaction motif (CIM) and is required for cIAP1/2 recruitment into TNF receptor complexes ^98^. Hence, as one might predict, the loss of TRAF2 may sensitize cells to death receptor mediated necroptotic killing by preventing cIAP1/2 recruitment and ubiquitylation of RIPK1 ^99, 100^. Remarkably, however, TRAF2 was recently reported to also exert anti-necroptotic activity independently of cIAP1/2 by binding directly to MLKL to limit the association of MLKL with RIPK3 ^100^. The steady state association of TRAF2 with MLKL was diminished upon TNF-induced necroptosis induction, and this correlated with CYLD dependent deubiquitylation of TRAF2. It will be interesting to define the TRAF2 interacting residues of MLKL, and whether their mutation impacts MLKL binding to RIPK3 and cellular necroptotic activity.

### Ppm1b

Ppm1b was recently identified as a phosphatase that dephosphorylates mouse RIPK3 at T231/S232 ^101^, two sites at which phosphorylation is known to enhance RIPK3 catalytic activity ^102^. While deficiency in Ppm1b led to a modest elevation in cell death in the absence of stimulation (5–10% *vs* 2–5% cell death, depending on cell line studied), the enhancement of cell death observed upon TNF-stimulation of L929 fibrosarcoma cells and TNF+pan-caspase inhibitor (zVAD-fmk) treatment of mouse embryonic fibroblasts was profound ^103^. This is the first illustration that necroptotic signaling can be tuned by dephosphorylation of RIPK3. It remains of outstanding interest whether there are other phosphatases that modulate the activity of RIPK3 and whether dephosphorylation of MLKL might serve as a mechanism to disarm its pro-necroptotic activity.

### A20

A20 is a deubiquitinating enzyme that is rapidly induced following TNF- and TLR-stimulation as part of a negative feedback loop to restrict NFκB activation and inflammatory cytokine production. Hence, its mutation or loss in mice and humans results in pronounced inflammatory disease. A20 has been proposed to restrict TNF-induced NFκB by removing K63-linked ubiquitin chains from RIPK1, and at the same time can target RIPK1 for proteasomal degradation through the addition of K48-linked ubiquitin chains ^103^. However, it also exerts its anti-inflammatory properties by targeting a number of TNF receptor and TLR signaling components ^104^. Recently it was found that A20 deletion in T-cells led to RIPK3 dependent T-cell killing, and that the perinatal lethality of A20 deficient mice was significantly rescued by RIPK3 co-deletion ^96^. Mechanistically, A20 loss promoted RIPK1-RIPK3 necrosome formation as a consequence of RIPK3 Lys5 ubiquitylation, while A20 expression and catalytic activity correlated with decreased RIPK3 ubiquitination.

## Open questions surrounding MLKL function

Because MLKL was only recently identified as an effector of the necroptosis cell death pathway, much remains to be garnered about how it functions in this pathway, whether it has additional “moonlighting” functions, and how it might contribute to other death signaling pathways. Should moonlighting functions exist for MLKL, they are likely very subtle, since genetic deletion of MLKL in mice does not noticeably impact viability, fertility or development ^58, 60^. The recent observation that, upon phosphorylation, MLKL translocates to the nucleus ^105^ suggests either a second (as-yet-unknown) function for MLKL in the nucleus or that the necroptosis signaling pathway relies on a transition *via* the nucleus to other membranes. This remains a matter of ongoing interest. The subcellular destination for MLKL that leads to cell death has also been a matter of debate: does MLKL-mediated death rely on translocation to mitochondrial or plasma membranes or another subcellular organelle? Initially, MLKL was implicated as being upstream of the mitochondrial phosphatase, phosphoglycerate mutase family member 5 (PGAM5), whose activation was thought to drive cell death through mitochondrial fragmentation *via* Dynamin-related protein 1 (Drp1) ^106^. However, several studies indicated that both PGAM5 and Drp1 are dispensable for necroptosis ^60, 107– 109^ and, as a result, the ubiquity of their involvement in the pathway has been questioned. Indeed, depletion of mitochondria by mitophagy did not prevent necroptotic death ^67^, suggesting that mitochondria are dispensable for necroptosis, at least in a subset of commonly studied laboratory cell lines. Nonetheless, reactive oxygen species (ROS), which emanate from mitochondria, have been widely implicated in necroptotic cell death ^59, 110– 112^. It remains to be determined whether ROS arise as a consequence of cell death or whether their generation is a driving force or augments necroptotic death and/or contributes to inflammatory responses. As described above, it is of enormous interest to understand the precise mechanism by which MLKL kills cells and whether other factors are involved downstream of MLKL phosphorylation and whether MLKL function can be modulated by ubiquitylation, as suggested by a recent study. Moreover, these modifications may govern whether MLKL can participate in other intersecting signaling pathways, such as inflammatory signaling in the absence of cell death ^18^ or cell death by pyroptosis.

## Abbreviations

4HB, four-helix bundle; CYLD, cylindromatosis; DAI, DNA-dependent activator of IFN-regulatory factors; DD, death domain; Drp1, Dynamin-related protein 1; FADD, Fas-associated protein with death domain; HOIP, HOIL-1 interacting protein; HSP90, heat shock protein 90; IAP, inhibitor of apoptosis proteins; IFN, interferon; LPS, lipopolysaccharide; LUBAC, linear ubiquitin chain assembly complex; MLKL, mixed lineage kinase domain-like; NFκB, nuclear factor kappa-light-chain-enhancer of activated B cells; PGAM5, phosphoglycerate mutase family member 5; PKR, protein kinase R; RHIM, RIP homotypic interaction motif; RIPK, receptor interacting protein kinase; ROS, reactive oxygen species; TLR, toll-like receptor; TNF, tumour necrosis factor; TNFR1, TNF receptor 1; TRADD, TNFR1-associated death domain; TRAF2, TNF receptor associated factor 2.
